# YAO is a nucleolar WD40-repeat protein critical for embryogenesis and gametogenesis in Arabidopsis

**DOI:** 10.1186/1471-2229-10-169

**Published:** 2010-08-11

**Authors:** Hong-Ju Li, Nai-You Liu, Dong-Qiao Shi, Jie Liu, Wei-Cai Yang

**Affiliations:** 1 Key Laboratory of Molecular and Developmental Biology, Institute of Genetics and Developmental Biology, the Chinese Academy of Sciences, Beijing 100101, China; 2 Gradute University, the Chinese Academy of Sciences, Beijing 100049, China

## Abstract

**Background:**

In flowering plants, gametogenesis generates multicellular male and female gametophytes. In the model system Arabidopsis, the male gametophyte or pollen grain contains two sperm cells and a vegetative cell. The female gametophyte or embryo sac contains seven cells, namely one egg, two synergids, one central cell and three antipodal cells. Double fertilization of the central cell and egg produces respectively a triploid endosperm and a diploid zygote that develops further into an embryo. The genetic control of the early embryo patterning, especially the initiation of the first zygotic division and the positioning of the cell plate, is largely unknown.

**Results:**

Here we report the characterization of a mutation, *yaozhe (yao)*, that causes zygote arrest and misplacement of cell plate of the zygote, leading to early embryo lethality. In addition, gametophyte development is partially impaired. A small portion of the mutant embryo sacs are arrested at four-nucleate stage with aberrant nuclear positioning. Furthermore, the competence of male gametophytes is also compromised. *YAO *encodes a nucleolar protein with seven WD-repeats. Its homologues in human and yeast have been shown to be components of the U3 snoRNP complex and function in 18S rRNA processing. *YAO *is expressed ubiquitously, with high level of expression in tissues under active cell divisions, including embryo sacs, pollen, embryos, endosperms and root tips.

**Conclusions:**

Phenotypic analysis indicated that *YAO *is required for the correct positioning of the first zygotic division plane and plays a critical role in gametogenesis in Arabidopsis. Since YAO is a nucleolar protein and its counterparts in yeast and human are components of the U3 snoRNP complex, we therefore postulate that YAO is most likely involved in rRNA processing in plants as well.

## Background

The model plant Arabidopsis forms multicellular male and female gametophytes, namely pollen grains and embryo sacs that contain the sperm cells and egg cell respectively. During embryo sac development, the functional megaspore, one of the four meiotic products, undergoes three consecutive rounds of mitotic division to produce an eight-nucleate embryo sac, which cellularizes simultaneously to form a seven-celled female gametophyte composed of one egg, two synergids, three antipodal cells and a diploid central cell [1-4]. The pollen grain in Arabidopsis is a tricellular structure that contains two sperms and a large vegetative cell. The vegetative cell germinates a pollen tube to deliver the two sperms to the female gametophyte for double fertilization that ultimately results in the formation of an embryo and endosperm.

Polarity and asymmetric cell division are a common feature of many different cell types, including the *Caenorhabditis elegans *zygote, the *Drosophila *oocyte and mammalian epithelial cells [5]. In the model species Arabidopsis, the fertilized egg or zygote undergoes a polarized elongation process to achieve an approximately 3-fold increase in length and apical localization of its nucleus prior to division. The first asymmetric zygotic division yields two cell lineages, namely a smaller apical and a larger basal cell, that adopt completely different developmental program. The apical cell goes through two rounds of vertical divisions and a subsequent round of horizontal division to form an octant embryo proper, but the basal cell undergoes repeatedly transverse division to form a linear suspensor that contributes to the quiescent centre of the root tip and connects the embryo to the maternal tissue.

In past decades, significant progress has been made in identifying genetic components controlling embryo development. The SeedGenes Project (http://www.seedgenes.org) has collected a large number of seed mutants, and provides phenotypic and molecular information on the essential genes in Arabidopsis [6,7]. It sets up a good foundation for large-scale and further analysis of the essential genes. Our knowledge about the genetic control of zygote development which establishes the apical and basal domain of the preglobular embryo is still quite limited although several mutations have been reported. In *yoda *(*yda*) mutant, the zygote fails to elongate properly and divides symmetrically resulting in incorporation of the basal lineage into the embryo proper. The gain-of-function mutation of *YDA *promotes basal cell lineage fate. This indicates that YDA acts as a switch between the two lineages [8]. YDA is a member of the MAPKK kinase family, which suggests that a MAP kinase signalling cascade is critical for the development of both apical and basal cell lineages. In *gnom/emb30 *mutants, zygotes are shorter than the wild-type and display aberrant zygotic division and also aberrant development from the first division stage on [9]. *GNOM *encodes an ARF GEF that controls endosomal trafficking and the polar secretion of auxin efflux carriers [10,11]. *EMBRYONIC FACTOR 1 *(*FAC1*), encoding an AMP deaminase, is essential for the zygote-to-embryo transition. In the *fac1 *mutant, embryo development is arrested at the single-celled zygote stage or the first zygotic division stage, forming a larger apical cell compared to that of the wild-type [12]. Similarly, in *root-shoot-hypocotyl-defective *(*rsh*) mutants, the position of the cell plate at the first division of the zygote results in a larger apical cell relative to the wild-type [13]. RSH is a hydroxyproline-rich cell wall glycoprotein essential for the correct positioning of the cell plate during cytokinesis in cells of the developing embryo. In the *ton/fass *mutants, occasionally the cell wall separating the apical from the basal daughter cell was oriented obliquely, instead of perpendicular to the axis [14]. All these findings suggest that a complicated gene regulatory network is involved during the zygotic elongation and cell division.

Recent studies revealed important roles of RNA processing and ribosome biogenesis in development including cell fate specification and cell cycle progression [15,16]. Proteomic analyses of human [17] and Arabidopsis [18] nucleoli revealed that there are many proteins related to cell cycle regulation, DNA damage repair and pre-mRNA processing present in the nucleolus. In Arabidopsis, several genes encoding nuclear spliceosome proteins were reported to play key roles in gametogenesis and embryogenesis. LACHESIS (LIS), GFA1/CLO/VAJ, and ATROPOS involved in mRNA splicing, play very important roles in gametic cell specification during embryo sac development [19-22]. MAGATAMA3 (MAA3), homologous to yeast SPLICING ENDONUCLEASE1 (SEN1), is required for central cell development and pollen tube guidance [23]. SWA1 and SWA2, involved in rRNA processing and ribosome biogenesis respectively, are required for cell cycle progression during embryo sac development [24,25]. *DOMINO1 *plays a role in ribosome biogenesis, and its loss-of-function mutation caused arrest of embryogenesis [26]. The recently reported AtRH36, involved in 18S pre-rRNA processing controls the mitotic division during female gametogenesis [27] TORMOZ (TOZ), a WD40 repeat domain-containing nucleolar protein plays important roles in orientating the division plane during early embryogenesis. In the *toz *mutant, the first division of the zygote is normal, but the longitudinal division planes of the apical cell are generally replaced by transverse divisions [28]. This is the first report that a nucleolar protein is required for positioning the division plane during early embryogenesis in Arabidopsis. It would be interesting to know whether such nucleolar proteins also control the positioning of the zygotic division plane.

Here, we describe a novel Arabidopsis mutant *yao *that affects the positioning of the zygotic cell division plane and gametophyte development. In *yao *mutant, zygote elongation and the correct cell plate positioning during early embryogenesis are impaired, a portion of the mutant embryo sacs are arrested at four-nucleate stage, furthermore, the competence of *yao *pollen is weaker than the wild-type. Molecular analysis shows that *YAO *encodes a conserved WD-repeat containing nucleolar protein that is homologous to hU3-55K/Rrp9, a component of the U3 small nucleolar ribonucleoprotein (snoRNP) in animals and yeasts. Our results indicate that YAO plays a key role in controlling the elongation and cell plane positioning of the zygote. It remains to be investigated how a nucleolar protein like YAO is involved in cell division plane of the zygote in Arabidopsis. Nevertheless, this work supports the earlier findings that RNA processing plays a critical role in embryo sac development and gametic cell fate in plants [20-26], although the precise mechanisms remain unknown.

## Results

### Genetic analysis of *yao *mutant

**Figure 1:**
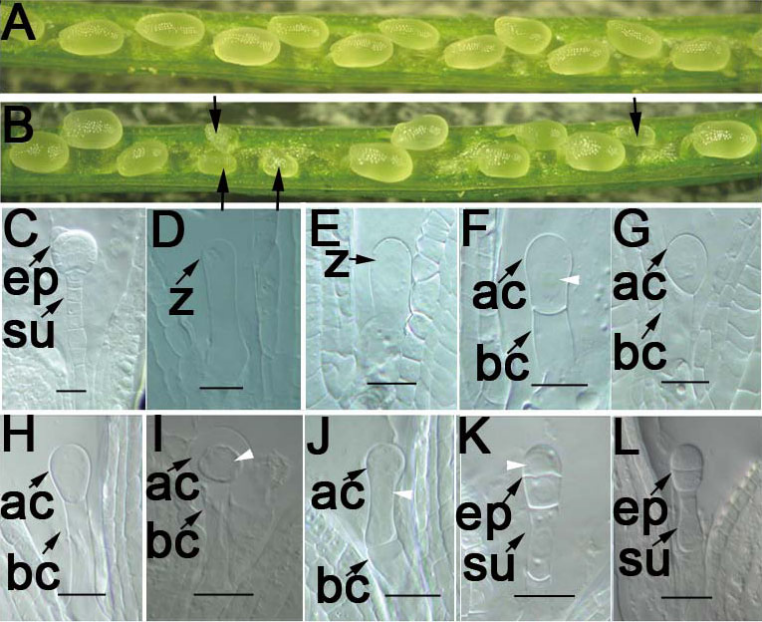
**Phenotype of the *yao *plant**. **A-B**: Photographs showing full seed set in wild-type (**A) **and partial seed set in *yao*/+ mutant siliques (**B**). **C-L**: Micrographs of cleared ovules from the same *yao*/+ silique showing the wild-type (+/+) embryo at the globular stage (**C**), and *yao/yao *mutant ovules (**D-L**). Mutant zygotes were either arrested (**D-E**) or divided once with misplaced division plane (**F-G**) and an enlarged apical cell (**H-J**). The abnormal apical cells divided transversely (**K-L**). **ac**, apical cell; **bc**, basal cell; **ep**, embryo proper; **su**, suspensor; **z**, zygote. Arrowheads indicating enlarged nucleoli. Bar = 20 μm.

To understand the molecular mechanisms that control gametophyte development in plants, we performed a genetic screen for mutants with distorted Mendelian segregation and reduced seed set in our gene/enhancer trap lines generated by *Ds *insertion [29-31]. A gene-trap line *yao *was isolated through this screen. Mutant plants, heterozygous for the *Ds *insertion, showed reduced seed set and displayed a kanamycin-resistant (*Kan*^*R*^) to kanamycin-sensitive (*Kan*^*S*^) ratio of 1.3:1 (*Kan*^*R*^/*Kan*^*S *^= 1156:885). Furthermore, these *Kan*^*R *^progenies were also defective in seed set. About 28% (n = 1637) ovules were small, shrunken, and finally aborted in heterozygous mutant plants. In contrast, full seed set was observed in wild-type siliques when grown in the same conditions (Fig. 1A and 1B). Together, the distorted *Ds *segregation and reduced seed set are indicative of defects in gametophyte and/or embryo development.

To define the embryo defects, cleared ovules from the same silique (*Ds/+*) were examined with a Zeiss Axioskop II microscope using differential interference contrast optics. The result showed that when the wild-type embryos reached the globular stage (Fig. 1C), the mutant embryos were arrested at zygote, one- or two-celled embryo proper stage. 6.68% zygotes (n = 443) were able to elongate but stopped developing further (Fig. 1D and 1E). 20% zygotes divided once with either symmetrical division plane resulting in two equally sized cells (Fig. 1F and 1J), or asymmetrically with an enlarged apical cell that could just enlarge but is unable to undergo subsequent longitudinal division (Fig. 1H and 1I). In some cases, the cell division plane was not perpendicular to the longitudinal axis of the zygote (Fig. 1G). And the apical cell was often ballooned with a large nucleolus and finally degraded as ovule collapsed (Fig. 1F, I, J and 1K). In a few cases (1.57%), the apical cells were able to divide not vertically but transversely, and the basal cell often went through one or two transverse cell divisions (Fig. 1K and 1L). In addition, it is common that the arrest embryonic cells contain a rather large nucleolus (Fig. 1E to 1L). Together, these observations showed that the mutation causes zygote arrest and disrupts the positioning of the zygotic division plane, suggesting that the gene disrupted by the *Ds *insertion plays an essential role in zygotic development.

To further investigate whether the mutation also affects male or female gametophyte function since the segregation of *Kan*^*R*^/*Kan*^*S *^deviated from the expected 2:1 ratio, the transmission efficiency of the mutation was determined by reciprocal crosses between the wild-type and the heterozygous mutant (*Ds/+*) plants. When the wild-type plant was used as pollen donor to pollinate mutant pistils (*Ds/+*), the F_1 _progeny displayed a *Kan*^*R*^/*Kan*^*S *^segregation ratio of 0.8:1 (361:450, n = 811, χ^2 ^= 4.6, p < 0.05), significantly deviating from the 1:1 ratio. This indicates that the mutation transmits through the female with a reduced transmission efficiency of 80%. When mutant plant was used as pollen donor to pollinate wild-type pistils, the *Kan*^*R*^/*Kan*^*S *^ratio is 0.58:1 (123:211, n = 334, χ^2 ^= 11, p < 0.05), with a significantly reduced transmission efficiency of 58% through the male. The above transmission efficiency through male and female predicted a 1.85:1 ratio of *Kan*^*R*^:*Kan*^*S *^in selfed progenies, which deviated significantly with the actual 1.3:1 ratio, suggesting that the mutation not only affects gametophytes but also caused embryo lethality. Consistently, mutant plants homozygous for the *Ds *insertion were never recovered, which suggests that the mutation caused embryo lethality.

**Figure 2:**
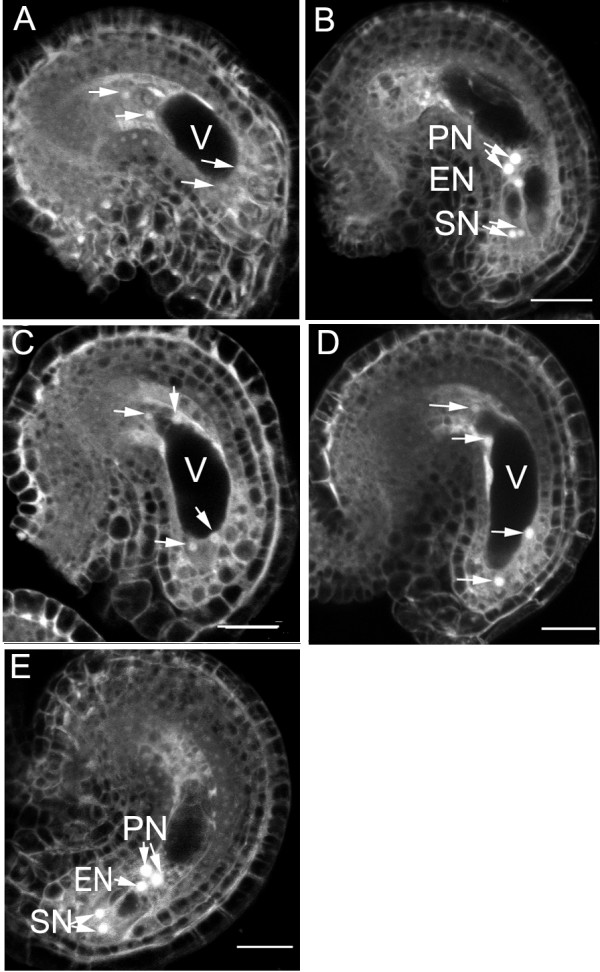
**Female gametophyte development in *yao/+ *siliques**. Confocal micrographs showing a female gametophyte at four-nucleate stage (FG4) (**A**) and FG5 stages (**B**) from wild-type plants, female gametophytes (**C-E**) from the same *yao/+ *pistil arrested at FG4 with misplaced nuclei at the micropylar pole (**C, D**) and reached to the FG5 stage (**E**). Compare the positioning of nuclei in **A **and **C-D**. **PN**, polar nucleus; **EN**, egg nucleus; **SN**, synergid nucleus; **V**, vacuole. Arrows indicating nucleus of the female gametophyte. Bar = 20 μm.

To investigate the female gametophyte (FG) phenotype, confocal laser scanning microscopy (CLSM) was used to compare embryo sac development in wild-type and the mutant as described previously [24,32]. In the wild-type, FG4 embryo sac contains 4 nuclei (Fig. 2A) and FG5 embryo sac contains two unfused polar nuclei (Fig. 2B). Flowers from both wild-type and mutant were emasculated at flower stage 12c [33], then the pistils were processed for CLSM analysis after 24 hr. At this time point, embryo sacs reached the seven-celled FG5 stage in the wild-type (Fig. 2B). In heterozygous mutant pistils, about 5% ovules (n = 251) were arrested at the four-nucleate FG4 stage (Fig. 2C and 2D), and 1% were at the two-nucleate FG3 stage. And the majority of the embryo sacs reached the FG5 stage, similar to that of the wild-type. The mutant embryo sacs often contain nuclei that are larger than that of the wild-type at the same stage (Fig. 2E), a phenomenon called nucleolar hypertrophy. Among the mutant embryo sacs arrested at FG4 stage, 80% showed an aberrant nuclear position at the micropylar pole (Fig. 2D) compared to the wild-type (Fig. 2A). These data showed that the majority of the mutant ovules developed normally, only a small fraction display arrested progression of the mitotic cycle. To confirm whether the defect of the mutant embryo sacs is caused by developmental arrest or delay, a delayed fertilization experiment was performed. We pollinated the *yao *mutant pistils 72 hr after emasculation with wild-type pollens, and the *Kan*^*R*^/*Kan*^*S *^rose from 0.8 to 0.89 (n = 231, χ^2 ^= 0.138, p > 0.05), which is not statistically significant. So the mutant embryo sacs were developmentally arrested. To further investigate if the mutant embryo sacs could be fertilized, mutant pistils (*Ds/+*) as egg donor were pollinated with wild-type pollen grains, and checked for embryo development. As a result, 94.4% (n = 500) ovules contained a zygote, and this was consistent with the observed 6% embryo sac arrest in the mutant. These data indicate that the mutation does have an impact on ovule development, but not severe.

Genetic analysis indicated that the mutation reduced pollen transmission significantly, however, no visible defects on the pollen development and pollen germination were observed, suggesting that the reduced transmission via male gametophytes was likely caused by a defect in pollen competence. To investigate this, we performed sparse pollination experiment where ovules were in excess. A small amount of mutant (*Ds/+*) pollen grains (< 30) were pollinated on wild-type pistils, and the F_1 _progenies were counted for *Kan*^*R*^/*Kan*^*S *^ratio. Indeed, the *Kan*^*R*^/*Kan*^*S *^ratio rose from 0.58:1 to 0.85:1 (n = 1661, χ^2 ^= 8.9, p < 0.05), indicating that more *yao *pollen can fertilize the ovule successfully when there is no competition of the wild type pollen. This suggests that YAO plays a role in pollen competence.

Together, these analyses indicate that the mutation caused a partial arrest of embryo sac development and reduced the competence of pollen. More importantly, the mutation impaired cell elongation and the positioning of the division plane of the zygote, which led to early embryo arrest.

### The mutant phenotype is caused by the *Ds *insertion in *YAO *gene

**Figure 3:**
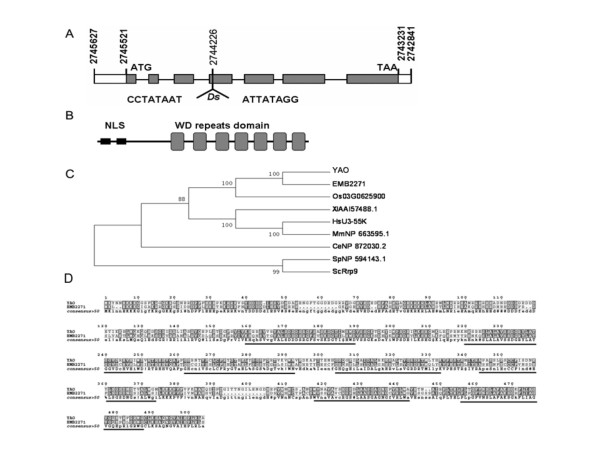
**Molecular characterization of the *YAO***. (**A**) Diagram of the *Ds *insertion in *YAO*. The grey boxes indicate the exons of *YAO*, the white boxes indicate the UTR regions and the lines indicate the intron regions. The nucleotide numbers are consistent with those in the Arabidopsis genome on TAIR web site. (**B**) Diagram of predicated domain structure of YAO protein. NLS, nuclear localization signal. (**C**) Phylogenetic tree of YAO with its homologs from other organisms: EMB2271 from *A. thaliana*; Os03G0625900 from *O. sativa*; HsU3-55K from *H. sapiens*; MmNP_663595.1 from *M. musculus*; XlAAI57488.1 from *X. laevis*; CeNP_872030.2 from *C. elegans*; SpNP_594143.1 from *S. pombe*; ScRrp9, from *S. cerevisiae. *(**D**) Protein alignment of YAO and EMB2271. The seven predicted WD40-repeat domains are underlined.

The genomic sequences flanking the *Ds *insertion was amplified by thermal asymmetric interlaced PCR and sequenced [34,35]. Analysis of the flanking sequences revealed that the *Ds *element was inserted into the fourth exon, 1395 bp downstream of the ATG initiation codon of *At4G05410 *gene, a gene of unknown function. The insertion resulted in 8 bp nucleotide duplication, typical for *Ds *insertion, at the insertion site (Fig. 3A).

To further determine whether the fertility phenotype that we observed is indeed the result of the *Ds *insertion within *At4G05410*, genetic complementation was performed. A 4600 bp genomic DNA fragment of *At4G05410*, including a 982 bp promoter region, 2293 bp coding region (including exons and introns) and 1325 bp 3'-UTR region, was amplified by PCR and cloned into pCAMBIA1301. The 1325 bp 3'-UTR region includes the minimal 3'-UTR region plus partial sequence of intron region next to the 3'-UTR of the next gene *At4G05400*. This construct was introduced into plants heterozygous for the *Ds *insertion by *Agrobacterium tumefaciens*-mediated floral dip method. After transformation, 33 independent transgenic T_1 _plants were obtained by double selection on Murashige and Skoog (MS) medium plates containing hygromycin and kanamycin. Among them, 30 transformants displayed obvious restoration of seed set, and five lines were then randomly chosen for a more detailed statistical analysis. Analysis showed that the ratio of normal to aborted seeds in T_1 _plants is close to 15:1 (n = 1197, χ^2 ^= 0.46, p > 0.49). In T_2 _generation, the *Kan*^*R*^/*Kan*^*S *^segregation ratio reached about 2.5:1, a significant increase compared to the 1.3:1 ratio in self-pollinated mutant plants. From four T_1 _transgenic lines, T_2 _plants homozygous for the *Ds *insertion, were recovered, indicating a full complementation of the transmission defect. Taken together, these data demonstrated that the genomic fragment fully complemented the mutation.

*YAO *encodes a polypeptide of 504 amino acids with an estimated molecular mass of 56.52 kDa, with seven WD-repeats and two nuclear localization signals (NLS) (Fig. 3B). A BLASTP search using the predicted YAO amino acid sequence showed that there are homologous sequences in other eukaryotic organisms, including *Arabidopsis thaliana*, *Oryza sativa, Xenopus laevis*, *Mus musculus*, *Saccharomyces cerevisiae*, *Schizosaccharomyces pombe*, *C. elegans *and *Homo sapiens. *Notably, YAO shares 63% identity and 75% similarity with EMB2271 (EMBRYO DEFECTIVE 2271, At4G21130) of Arabidopsis, 52% identity and 71% similarity with Os03G0625900 of rice, 37% identity and 56% similarity with Rrp9 of yeast, and 36% identity and 56% similarity with hU3-55K of human. Interestingly, a single copy of *YAO *gene is present in other organisms, unlike that in Arabidopsis where a twin homologue exists [36]. Phylogenetic analysis showed that YAO is more close to EMB2271 than others including the rice homologue (Fig. 3C and 3D), suggesting that they may be functionally conserved.

Both yeast Rrp9 and human hU3-55K had been studied extensively and were shown to be localized in nucleolus and play an essential role in 18S rRNA maturation. Rrp9/hU3-55K protein is a component of the U3 snoRNP complex that controls the early cleavages of the pre-rRNA transcripts at A_0_, A_1_, and A_2 _sites [37]. The WD-40 repeat domain of Rrp9/hU3-55K is involved in physical association with Snu13/15.5k and the snoRNA box B/C motif, and the complex formed is crucial for cell growth [37-39]. Rrp9/hU3-55K is an essential protein for cell viability, so is YAO in Arabidopsis. These suggest that YAO may play a similar role in rRNA biogenesis in Arabidopsis.

### YAO is a nucleolar protein

**Figure 4:**
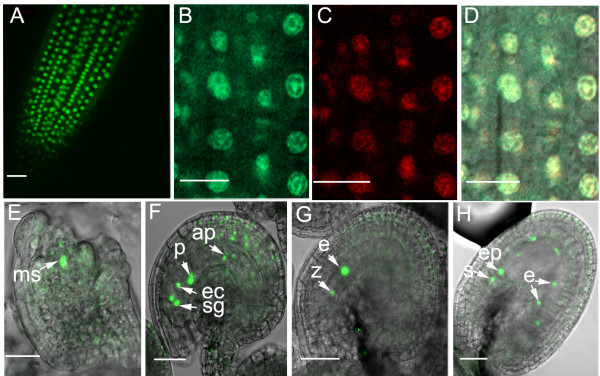
**YAO is localized in the nucleolus**. (**A**) Confocal micropgraphs showing nuclear localization of YAO-GFP in root tip. (**B-D**) Confocal micrographs of the same cell showing the subcellular localization of YAO-GFP (**B**), nucleolar marker SWA2-DsRed (**C**), and the merged image (**D**), note the nucleolar localization of YAO. (**E-H**) Confocal micrographs showing YAO-GFP localization in megaspore mother cell (**E**), embryo sac and integument cells (**F**), zygote and endosperm (**G**), endosperm and preglobular embryo (**H**) nuclei. **ac**, apical cell; **ap**, antipodal cell; **bc**, basal cell. **e**, endosperm; **ec**, egg cell; **ms**, megaspore mother cell; **pn**, polar nucleus; **sg**, synergid; **z**, zygote. Bar = 20 μm.

YAO is predicted to contain two N-terminal nuclear localization signals. To define its precise subcellular localization, a C-terminal translational fusion of YAO with GFP driven by the *YAO *native promoter was cloned into pCAMBIA1301. The *pYAO*::*YAO-GFP *construct was subsequently transformed into both wild-type and mutant plants. The seed set was increased significantly in T_1 _mutant plants transformed with *pYAO*::*YAO-GFP *and the construct completely complemented the mutant phenotype, indicating that the YAO-GFP fusion protein is functional in planta. Then, confocal laser scanning microscopy was performed on the wild-type plants expressing the *pYAO*::*YAO-GFP *fusion gene. YAO-GFP fluorescence was detected in the nucleus of *pYAO*::*YAO-GFP *transformed root cells and co-localized with a nucleolar marker SWA2-DsRed (Fig. 4A-D) [25]. Consistent with the mutant phenotype, strong GFP signal was detected in megaspore mother cell, female gametophytic cells, embryo and endosperm (Fig. 4E-H). YAO-GFP can also be detected in the integument with a relatively low level. These indicate that YAO is indeed a nucleolar protein which is consistent with its putative role in pre-rRNA processing.

### The *YAO *gene is preferentially expressed in tissues active in cell division

**Figure 5:**
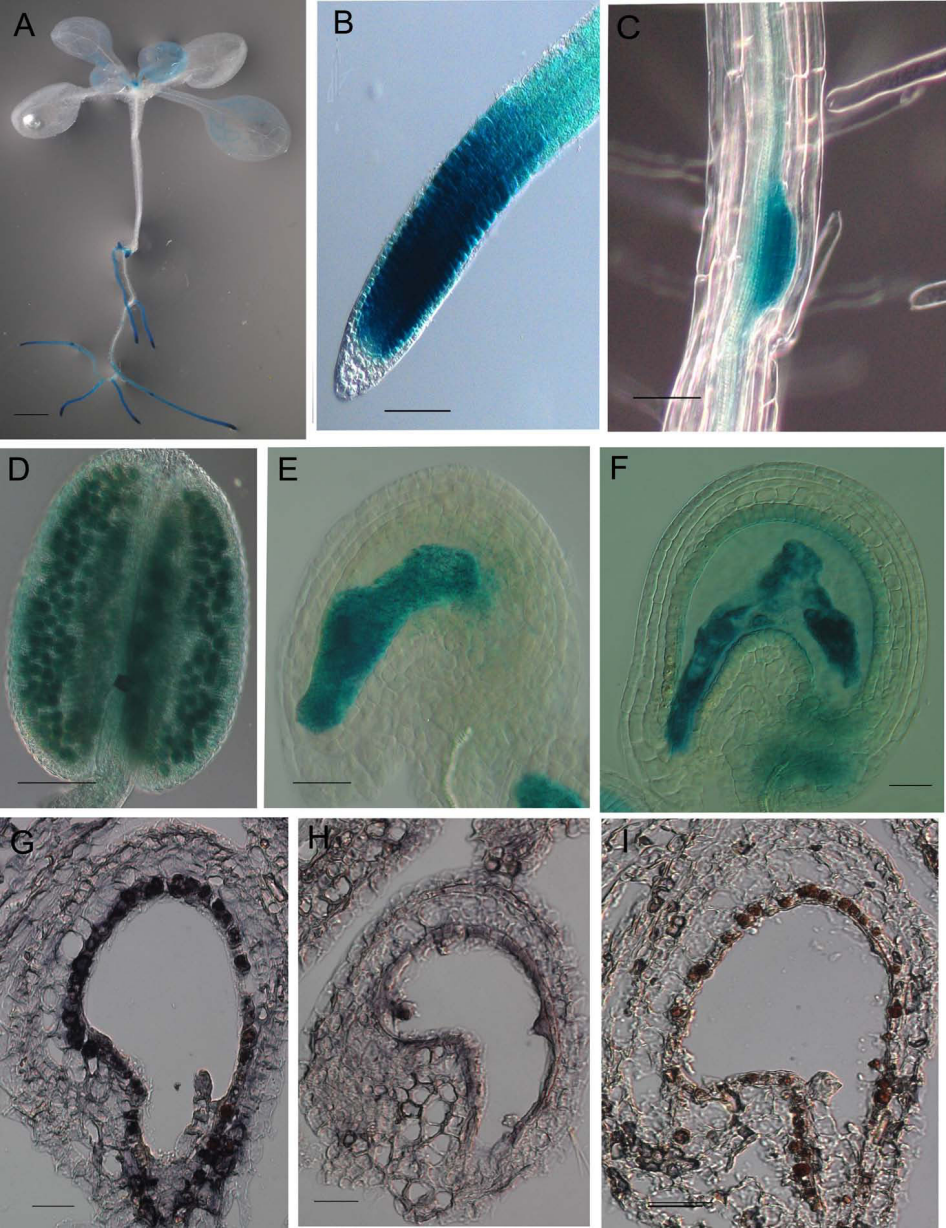
**Expression pattern of *YAO***. GUS staining showing *YAO *expression in a 10-day-old seedling (**A**), root tip (**B**), and lateral root primordial (**C**), pollen grains (**D**), embryo sacs before fertilization (**E**), and embryo and endosperm (**F**). RNA in situ showing *YAO *mRNA in early embryo (**G**) and endosperm (**H**), and sense control (**I**). Bar: 1 mm (**A**), 20 μm (**E-I**), 50 μm (**C, D**), 100 μm (**B**).

To investigate *YAO *cellular expression pattern, we used a *pYAO*::*GUS-3U *to monitor expression in plant tissues. A 982 bp fragment upstream from ATG start codon plus 30 bp of the first exon of *YAO *was used to drive the *GUS *reporter gene. In addition, the 1331 bp 3'-UTR fragment immediately downstream of the stop codon was also cloned behind the *GUS *coding sequence. Eight independent transgenic lines were analyzed. In all cases, high level of GUS activity was detected in tissues that are active in cell division, including shoot apexes, root tips and lateral root primordia, embryos and endosperm, pollen grains, embryo sacs (Fig. 5A-F). GUS signal in the integument is very low, which can only be detected under dark field microscopy (not shown). This is consistent with the YAO-GFP data which show YAO expression in the integuments. GUS activity is dynamic during embryogenesis, strong in early embryos, and very weak in globular embryos and thereafter hardly detectable. Furthermore, RNA in situ hybridization with specific probe of *YAO *gene showed that *YAO *is expressed in early embryo and endosperm (Fig. 5G-I). The microarray data available at the GENEVESTIGATOR (http://www.genevestigator.ethz.ch) also confirmed the expression profile revealed by the RNA in situ hybridization and promoter-GUS assay.

In summary, *YAO *is expressed preferentially in both gametophytes and early embryos and endosperms during embryogenesis. This expression pattern is in agreement with its essential role during embryogenesis. In addition, its high expression in tissues active in cell division suggests that *YAO *plays an important role in actively dividing cells during vegetative development.

## Discussion

### *YAO *is critical for gametogenesis and early embryo development

Genetic studies indicated that *YAO *plays an important role for gametophyte development and is critical for the division of zygote and the apical cell. Both male and female gametophytes are partially affected by the *yao *mutation. During early embryogenesis, the first division of zygote and the apical cell lineage were impaired in the mutant. This clearly indicates a critical role of *YAO *function in zygotic development and the patterning of the apical cell lineage, although the precise mechanism remains to be revealed. A limited number of genes controlling zygotic division, especially the decision of cell plate position are reported. In this aspect, *yao *is a novel mutation that acts in early embryogenesis. The pleiotropic and partial developmental arrests of ovules and embryos suggest that *YAO *may function at multiple stages during plant reproduction. In *yao/+ *selfed progenies, 28% ovules were defective which include developmental arrests of embryo sac, zygote and the apical cell lineage. Furthermore, the mutation did not affect pollen development and pollen germination *in vitro*; rather it impaired the competence of the male gametophyte. The weak competence of *yao *pollen might be caused by slow pollen tube growth.

The high level of sequence homolgy between YAO and EMB2271 proteins suggests that they may be functionally homologues. However, loss of function of *EMB2271 *displayed a late embryo-defective phenotype (SeedGenes database: http://www.seedgenes.org/). This could be simply caused by a difference in their temporal expression pattern. Indeed, *EMB2271 *is expressed only after globular stage, no expression could be detected before globular stage when *YAO *is expressed (Additional file 1). Their complementary expression pattern support the idea that *YAO *could function during early embryogenesis and *EMB2271 *during late embryogenesis.

In *yao *mutant embryo sac and embryos, the nucleolus is often larger than that in the wild-type, indicating they may be defective in nucleolar structure. Nucleolar hypertrophy has been reported in Arabidopsis [26,40-42], and linked to defect in ribosome biogenesis. Inactivation of nucleolus protein AtLA1 causes block of embryogenesis at the globular stage with hypertrophic cells [40]. Nucleolar machineries organize differently when ribosome biogenesis is inhibited [43,44]. Therefore, the nucleolar hypertrophy in *yao *embryo sac, zygote and preglobular embryos may imply a defect in ribosome biogenesis. Furthermore, Lee and colleagues reported a subset of WD-repeat containing proteins including YAO, in which, they confirmed 11 that interact with DDB1 and serve as substrate receptor of CUL4-DDB1 machinery [45]. It would be interesting to investigate whether YAO is also degraded by the CUL4-DDB1 machinery; nevertheless, we did not detect direct interaction between WD-repeat domain, full length YAO and DDB1 (DDB1a and DDB1b) by yeast two-hybrid assay. So YAO may not be component of CUL4-DDB1 complex.

### YAO is a nucleolar protein and likely involved in 18S pre-rRNA processing

Rrp9/hU3-55K is a non-ribosome nucleolar protein that is present in 90S pre-ribosome essential for 18S rRNA maturation and 40S subunit biogenesis [46]. The U3 snoRNP is composed of a small nucleolar RNA (snoRNA) and other non-ribosome proteins. Binding of Rrp9/hU3-55K to the U3 snoRNA B/C box is essential for pre-rRNA processing and cell growth [47]. The WD-repeat domain of the hU3-55K protein is required for its association with the U3 snoRNA and 15.5K protein [48]. In Arabidopsis, YAO and EMB2271 are the two proteins that are most homologous to hU3-55K and Rrp9, and YAO is more similar to hU3-55K in amino acid sequence. Its protein domain structure, nucleolar localization, and homology to hU3-55K and Rrp9, together make it likely that YAO is also a putative U3 snoRNP component involved in 18S pre-rRNA processing.

To investigate the interaction between YAO and 15.5 K counterpart in Arabidopsis (At5G20160 and At4G22380) using the yeast two-hybrid assay, no interaction between them was detected (data not shown). Their physical interaction may need the U3 snoRNA or a tethering factor as shown in human cells [38,39]. A pull-down assay to check the physical interplay of YAO, 15.5 K counterpart and the U3 snoRNA in Arabidopsis may help to elucidate the conserved function of YAO. In addition, TOZ and SWA1 are the putative components of the U3 snoRNP complex involved in rRNA processing in Arabidopsis. They are required for embryo sac development [24] and the correct positioning of the division plane during early embryogenesis [28], respectively. Although there is no direct physical interaction between YAO, TOZ and SWA1 in the yeast two-hybrid assay (data not shown), it is still possible that they share the same core components of the U3 snoRNP complex. In this respect, a proteomic approach would be useful to identify the U3 snoRNP components as done in yeast cells.

### Nucleolus and reproductive development

Embryo sac development and embryogenesis in plants require coordinated, rapid cell growth and proliferation, therefore active transcription and translation would be required to sustain rapid cell growth. In this respect, ribosome biogenesis, a key function of the nucleolus, is vital to these processes. It may be not surprising those mutations in genes that are involved in mRNA and pre-rRNA processing, ribosome biogenesis, cause developmental arrest of embryo sacs and embryos. These include *LIS*, *GFA1*/*CLO*/*VAJ*, *ATROPOS*, and MAA3 in mRNA splicing [19-22], SWA1 and *AtRH36 *in pre-rRNA processing [24,27], *SWA2 *and *DOMINO1 *in ribosome biogenesis [25,26]. *TOZ*, encoding a WD40 repeat domain-containing nucleolar protein plays important roles in orientating the division plane during early embryogenesis [28]. Whether TOZ is also involved in RNA processing or ribosome biogenesis is not known. Here we show that *YAO *also encodes a nucleolar protein with seven WD40-repeats and likely involved in rRNA processing as well. Loss of *YAO *function also impairs the correct positioning of the cell division plane in zygote and preglobular embryos. Are these just coincidences? Or they reflect a key role of the nucleolus in cell growth and proliferation during reproductive development. Embryo sac development is a unique process in which nuclear divisions are not immediately followed by cytokinesis, instead cytokinesis takes place simultaneously only after the completion of three rounds of nuclear division. In addition, the cytoplasm of the embryo sac is more or less separated to two poles by a large central vacuole and is a polar structure. These features may allow the embryo sac to use the nucleolus, i.e. ribosome biogenesis, as a central controller for its rapid growth and proliferation.

Another scenario may be that there is a regulatory role of the nucleolus other than a site of ribosome biogenesis as conventionally thought. There are reports that suggest nucleolar proteins play direct roles during cell cycle. CDC14 is trapped to the nucleolus by Net1 during G1 phase of the cell cycle, and is released and becomes active when cells enter into mitosis, leaving the Net1 stays in the nucleolus [49,50]. Here, the nucleolus acts as a sequestering compartment for regulatory complexes. The U3 complex-associated proteins shuttle between the nucleus and cytoplasm provide them an opportunity for their regulation and their interaction with other regulatory proteins [51]. YAO-GFP fusion protein is unevenly distributed in nucleolus, which is a new discovery and may be clue to explore the molecular and cellular function of YAO. Although a previous high through-put study on Arabidopsis nucleolus proteins showed that YAO is localized in nucleolus and small nuclear bodies reminiscent of Cajal bodies and speckles in Arabidopsis cultured cells [18]. But we never observed the Cajal bodies or speckle-like localization of YAO. In this report, the use of native promoter, fusion construct and functional assay make the YAO-GFP localization more precise and closer to the native condition. Further studies on the specific distribution of YAO in the nucleolus and the dynamic localization during cell division may help to elucidate the function of nucleolus in plant cell division.

## Conclusions

In conclusion, YAO is a nucleolar protein with seven WD40-repeats that plays a role in embryo sac development and is critical for the correct positioning of the division plane of zygote and the apical cell lineage in Arabidopsis. It is likely that YAO acts by modulating nucleolar function, such as rRNA biogenesis during gametogenesis and early embryogenesis. Further dissection of YAO function and the identification of its interacting proteins would shed light on how YAO regulates cell divisions during early embryogenesis in plants.

## Methods

### Plant material

Seeds of *Arabidopsis thaliana *ecotype Landsberg *erecta *were sterilized with 20% (v/v) bleach for 5-10 min, then washed five times in sterilized water and germinated on MS agar plates. For antibiotic selection, 50 mg/L kanamycin and/or 20 mg/L hygromycin were supplemented as required. Plants were grown in growth room at 22 ± 2 under a 16-hr-light/8-hr-dark cycle. Arabidopsis transformation was performed via floral dip method [52].

### Molecular analysis

The *Ds *flanking sequences were isolated by thermal asymmetric interlaced PCR as described previously [34,35]. To conduct the complementation experiment, A 4600 bp genomic DNA fragment encompassing from 982 bp upstream of the ATG initiation codon to 1325 bp downstream of the TAA stop codon of *At4G05410 *was amplified by KOD-plus polymerase (TOYOBO) using primers YAO-F (5'-TCAGCTGCAGACAAATAGAGGTAGGGGGAGAGTT-3'), and YAO-R (5'-GACACTGCAGCCGGCGAATCGAGGTATGG-3'), and cloned into pCAMBIA1301 (http://www.cambia.org.au).

For *pYAO::YAO-GFP *fusion construct, a 763 bp EGFP coding sequence was obtained from pEGFP (Clontech) by *Kpn*I and *Xba*I digestion, and subsequently cloned into pCAMBIA1301 to yield p1301-GFP construct. A 1325 bp fragment downstream the stop codon was first amplified by PCR with primers YAO-3'UTR-F (5'-ATCGTCTAGAGCATAAGTATTTCATTGGG-3') and YAO-3'UTR-R (5'-ACTGTCTAGACCGGCGAATCGAGGTATGG-3'), and subcloned into p1300-GFP to obtain *p1301-GFP-3'U *construct. A 3275 bp fragment containing a 982 bp promoter and the coding region with the TAA stop codon deletion was cloned to *p1301-GFP-3'U*, to give rise to the *pYAO::YAO-GFP *construct.

*For pYAO::GUS-3U *construction, a 1331 bp fragment from the stop codon TAA downstream is amplified with primers YAO-3'UTR-F (5'-ATCGGAGCTCGCATAAGTATTTCATTGGG-3') and YAO-3'UTR-R (5'-ACTGGAGCTCCCGGCGAATCGAGGTATGG-3'), and cloned into pBI101 to yield *pBI101-3U*. Then, a fragment including 1012 bp (-982 bp to +30 bp) was amplified and cloned into *pBI101-3U *to yield the *pYAO::GUS-3U *construct.

### Bioinformatic analysis

We used NCBI (http://www.ncbi.nlm.nih.gov/) to analyze the cDNA and genomic sequence DNA and DNAMAN and MEGA2 software to perform the phylogenetic analysis.

### Microscopy

To determine the embryo phenotype, siliques from *YAO*/+ plants were dissected with hypodermic needles and cleared in Herr's solution containing lactic acid:chloral hydrate:phenol:clove oil:xylene (2:2:2:2:1, w/w) [53]. Embryo development was studied microscopically with a Zeiss Axioskop II microscope equipped with differential interference contrast optics.

GFP and DsRed [25] images are captured with a LSM-510 META confocal laser scanning microscope (Zeiss, Yena, Germany). The GFP and DsRed were excited using the 488 nm and 543 nm laser respectively.

For GUS staining assay, samples were stained in 100 mM sodium phosphate buffer, pH 7.0, 0.1% Triton X-100, and 1 mg/mL X-Gluc, 2 mM potassium ferricyanide and potassium ferrocyanide. The samples were vacuum-infiltrated in X-Gluc solution for 10 min and kept at 37°C for 4 hours, then cleared in 30% lactic acid and 20% glycerol, and finally observed with a Zeiss Axioskop II microscope.

### RNA in situ hybridization

For RNA labelling, *YAO *cDNA fragment was amplified using primer YAO-insitu-F (5'-GATGCGGAAGAGAACGGATTTA-3') in combination with YAO-insitu-R (5'-CACTTGGCCATATGTATTTGTCAG-3') and cloned into PGEM-Teasy vector (Promega) to produce *pGEM-T Easy*-*YAO*. It is linearized by *Nco*I digestion for antisense RNA probes and by *Sal*I digestion for sense RNA probes. Antisense and sense probes were transcribed *in vitro *with SP6 and T7 RNA polymerase, respectively, and labelled using the digoxigenin RNA labeling kit (Roche). Prehybridization, hybridization, washing, antibody staining, and signal detection were performed as described previously[24].

## Authors' contributions

HJL and NYL carried out the experimental studies data acquisition. HJL performed the analysis and interpretation of data. HJL and WCY conceived, coordinated the study and drafted the manuscript. DQS and JL provided technical assistance. All authors gave ideas, revised, read and approved the final manuscript.

## Supplementary Material

Additional file 1 **Comparison of *YAO *and *EMB2271 *expression in Arabidopsis revealed by RT-PCR analysis**. RNAs were extracted from pistils before (**P1**) and 24 hr after pollination (**P2**), and siliques in which the embryo is at globular (**G**), late globular (**LG**) or cotyledon (**C1**, **C2**) stages, leaf (**Lf**) and inflorescences (**In**). RNAs were reverse-transcribed and amplified by PCR with primer combinations YAO-F (5'-CAGCTTCTTCCGTCGCCACTAAAC-3')/YAO-R (5'-CTCCCATCTTCATCTCCGCCAG-3') and EMB2271-F (5'-GAAGTTTTGAAGTCTCAC-3')/EMB2271-R (5'-CGATAGATCAACCGAGTAG-3'), respectively. Note: *YAO *is expressed in pistils before and shortly after fertilization, but not in siliques at globular and cotyledon embryo stages, while *EMB2271 *is only weakly expressed in siliques at globular and early cotyledon embryo stages. *eIF4A *was used as an internal control. Genomic DNA (**Ck**) was used as control to verify primer combination and PCR amplification. Click here for file
