# Supplementary figures and images for: YAO is a nucleolar WD40-repeat protein critical for embryogenesis and gametogenesis in Arabidopsis
Source: BMC Plant Biol. 2010 Aug 11;10:169. doi: 10.1186/1471-2229-10-169 (PMC3095302; doi:10.1186/1471-2229-10-169)

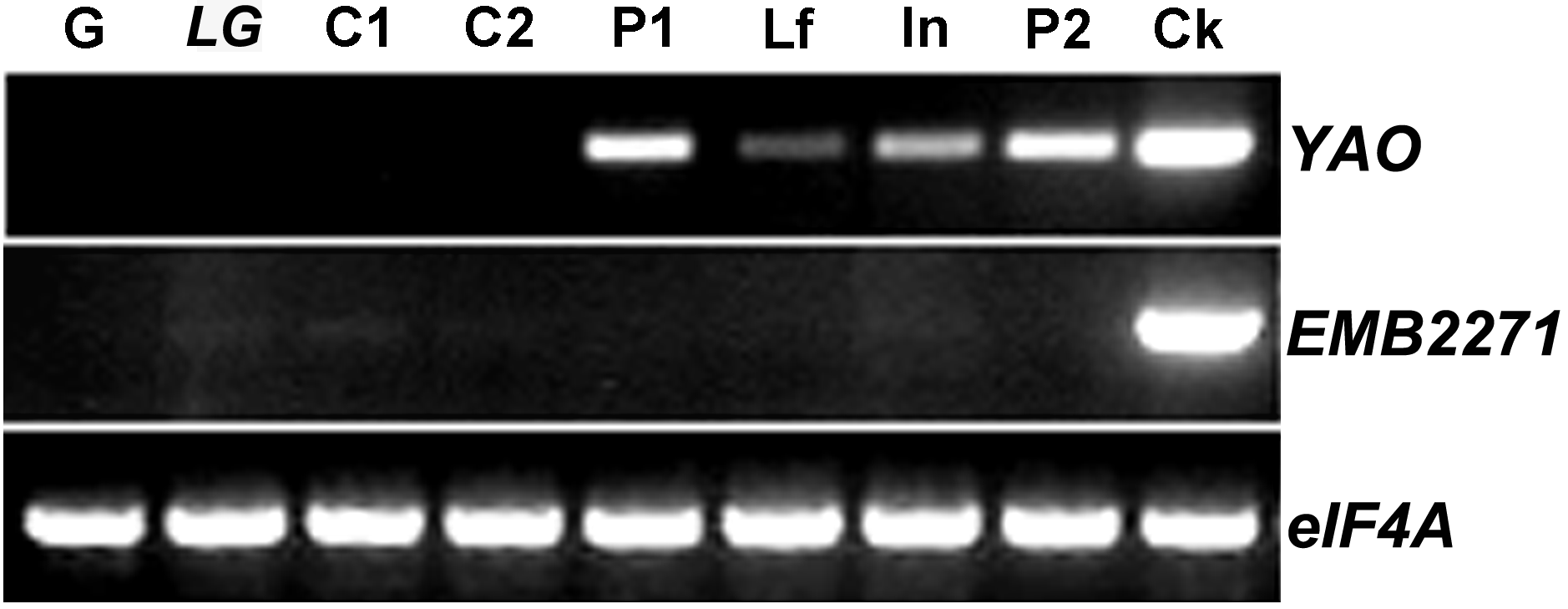

Supplement: Additional file 1 — Comparison of YAO and EMB2271 expression in Arabidopsis revealed by RT-PCR analysis. RNAs were extracted from pistils before (P1) and 24 hr after pollination (P2), and siliques in which the embryo is at globular (G), late globular (LG) or cotyledon (C1, C2) stages, leaf (Lf) and inflorescences (In). RNAs were reverse-transcribed and amplified by PCR with primer combinations YAO-F (5'-CAGCTTCTTCCGTCGCCACTAAAC-3')/YAO-R (5'-CTCCCATCTTCATCTCCGCCAG-3') and EMB2271-F (5'-GAAGTTTTGAAGTCTCAC-3')/EMB2271-R (5'-CGATAGATCAACCGAGTAG-3'), respectively. Note: YAO is expressed in pistils before and shortly after fertilization, but not in siliques at globular and cotyledon embryo stages, while EMB2271 is only weakly expressed in siliques at globular and early cotyledon embryo stages. eIF4A was used as an internal control. Genomic DNA (Ck) was used as control to verify primer combination and PCR amplification. [file 1471-2229-10-169-S1.TIFF]
